# Urban-rural disparity in cervical cancer in China and feasible interventions for tackling the rural excess

**DOI:** 10.1097/MD.0000000000013907

**Published:** 2019-01-04

**Authors:** Xiaoduo Wen, Denggui Wen, Yi Yang, Yuetong Chen, Kohei Akazawa, Yunjiang Liu, Baoen Shan

**Affiliations:** a Gynecological and Obstetrical Ultrasound; b Cancer Center, The Fourth Hospital of Hebei Medical University, Shijiazhuang, China; c Medical Information, Affiliated Hospital, Niigata University, Niigata, Japan.

**Keywords:** changing sex behavior, population-based tumor registration, screening, urban-rural disparity in cervical cancer

## Abstract

According to GLOBOCAN 2012, age-standardized incidence rate (ASIR) of cervical cancer in developed and less developed countries is 9.9 vs. 15.7 per 100,000 population per year. This disparity is related to inequity in access to screening. Urban rural disparity in access to cervical cancer screening is similar in China. We aim to assess urban rural disparity in ASIR.

Using population-based tumor registration data collected by us in urban Shijiazhuang city (with incidence data available for 1,217,437 women in 2012) and in Shexian County (with incidence data available for 197,416 women since 2000), we compared ASIR of cervical cancer between the two populations in 2012. We also analyzed the trend of biennial ASIR and averaged age at diagnosis of cervical cancer for 2000–2015 in Shexian County during which China was undergoing rapid changes in sexual mores. Finally, using previously published national death survey data, we compared age-standardized mortality rate (ASMR) of cervical cancer between Shijiazhuang city and Shexian County over the periods of 1973–1975 and 1990–1992.

It was found that the ASIR of cervical cancer in rural Shexian County is 3 times higher than in Shijiazhuang city in 2012 (25.0 vs. 8.4 per 100,000 per year, *P* < .01); and the corresponding ASMR was 2 times higher over the period of 1973–1975 (25.0 vs. 13.0 per 100,000 per year, *P* < .01) and 8 times higher over the period of 1990–1992 (9.8 vs. 1.2 per 100,000 per year, *P* < .01). From 2000 to 2015 along with rapid changes in sexual behavior, the biennial ASIR of cervical cancer increased by +3.2% on average, from 19.3 to 28.5 per 100,000 per year (*P* < .01), and the biennial averaged age at diagnosis decreased from 55.8 to 52.1 (*P* < .01).

Urban-rural disparity in ASIR of cervical cancer in present study is larger than that reported between developed and less developed countries in GLOBOCAN 2012, in which the disparity is considered “due to differences in access to screening.” As in China, cytologists and infrastructure required for cervical cancer screening are similarly lacking in rural areas, we suggest cytological screening for cervical cancer be strengthened in disadvantaged rural settings.

## 1 Introduction

According to GLOBOCAN 2012, worldwide there were 528,000 new cervical cancer cases and 266,000 deaths. Eighty-five percent of new cases and 87% of deaths were in low and middle income countries (LMICs). Significant difference in age-standardized incidence rate (ASIR) of cervical cancer exists between developed and less developed countries (9.9 vs. 15.7 per 100,000 per year), due to differences in access to screening and health services.^[1,2]^ Urban-rural disparity in social determinants of health in China is comparable to that observed between developed and less developed countries. However, urban-rural difference in ASIR of cervical cancer reported so far remains controversial. For example, according to the statistics released by Chinese National Cancer Registration Center (CNCRC), from 1989 to 2008, the biennial ASIR of cervical cancer (per 100,000 per year) for rural women was 1.9–8.9, about the same of urban women of 2.3–8.1.^[3]^ Based on data in 14 prefecture cities and 18 counties, between 2003 and 2007, the five-year rural versus urban ASIR of cervical cancer is 7.5 vs. 6.6 per 100,000 per year^[4]^; and in the following years of 2009, 2010, 2011, 2012, and 2013, the annual rural-urban ASIR of cervical cancer is 8.9 vs. 9.1, 8.8 vs. 9.6, 9.4 vs. 9.7, 9.9 vs. 10.5, and 10.5 vs. 10.1 per 100,000 per year, respectively.^[5–9]^ To clarify the issue, we compared ASIR of cervical cancer between rural Shexian County and urban Shijiazhuang city using population-based tumor registration data collected by us in Shijiazhuang city (2012) and Shexian County (2000–2015), and retrospectively analyzed the difference in age-standardized mortality rate (ASMR) between the two places using previously published national death survey data.

## 2 Materials and methods

A cross-sectional study is performed to assess urban-rural disparity in incidence and mortality rate of cervical cancer over different time periods. First ASIR of cervical cancer in 2012 was compared between rural Shexian County and urban Shijiazhuang city. We chose 2012 for comparison because population-based tumor registration data (incidence data) in Shijiazhuang city are available only for 2012, but in Shexian County, the data are available since 2000. We then compared ASMR of cervical cancer between the two populations over the periods of 1973–1975 and 1990–1992 using previously published national death survey data. Finally, we examined the increasing trend of ASIR of cervical cancer in Shexian County from 2000 to 2015 during which China has been undergoing rapid changes in sexual behavior in parallel with socioeconomic development and urbanization.

### 2.1 Background of Shexian County and Shijiazhuang city

**Figure 1:**
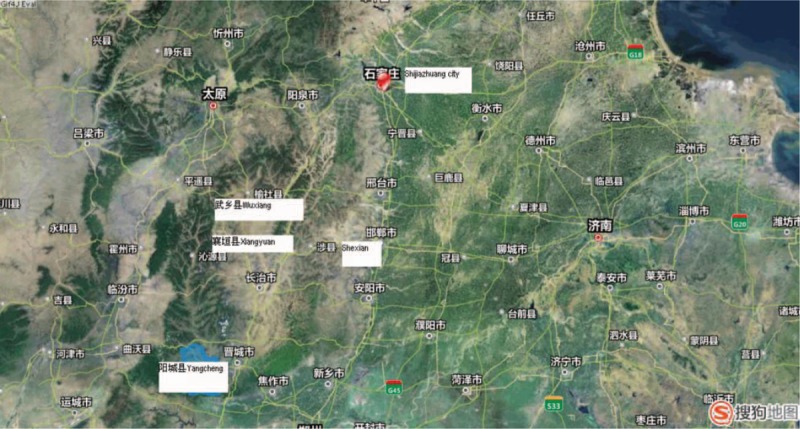
Geographic location of urban Shijiazhuang city, rural Shexian County, and the adjoining Xiangyuan, Wuxiang, and Yangcheng counties in Shanxi province where endemic rates of cervical cancer are found.

Shexian County is located 250 km south west of Shijiazhuang city in Hebei province (Fig. 1). The county extends from 36°17′ to 36°55′ N latitude along the Taihang Mountain range, which divides the Shanxi and Hebei province. Shexian is adjoining Xiangyuan, Wuxiang, and Yangcheng counties of Shanxi province in the west. All these counties had endemic rates of cervical cancer in the 1973–1975 first national morality survey.^[10,11]^

Shijiazhuang is the capital city of Hebei province, located 270 km south of Peking, at the crossing point of north-south-bound Peking-Wuhan railway and the east-west-bound Shijiazhuang-Taiyuan railway. Shijiazhuang has not been considered as an urban city before 1953. It consisted of several dozens of villages. Its urbanization began as late as 1953, when it was chosen by the Chinese central government as the capital city of Hebei province.

### 2.2 Data sources of incidence rate of cervical cancer by population-based cancer registration in Shexian County and Shijiazhuang city

Population-based cancer registration in Shexian County was established in 1999 and in Shijiazhuang city in 2011, under the agreement of Chinese National Cancer Registration Center (CNCRC) and International Association of Cancer Registration (IACR). Tumor registration in the two places is run by us (the same team) according to the principles of IACR. Cancer diagnoses are reported to registries from multiple sources, including local hospitals and community health centers, as well as the Urban Resident Basic Medical Insurance program and the New Rural Cooperative Medical Scheme, both of which are public insurance programs with universal coverage of local registered urban or rural residents achieved by 2010. Variables collected in tumor registration include patient ID, birth date, sex, age at diagnosis, bases of diagnosis, topography, histology, and survival status. Because stage at diagnosis was not collected, the effect of screening cannot be assessed. Data quality of cancer registration is assessed annually by CNCRC before publication. On July 21, 2017, the incidence data of Shexian Cancer Registry for 2008–2012 were accepted by IACR for inclusion in the “Cancer Incidence in 5 Continents Vol. XI”.

### 2.3 Data sources of cervical cancer mortality in Shijiazhuang city and Shexian County

China conducted three national retrospective mortality surveys over the periods of 1973–1975,^[10,11]^ 1990–1992,^[12]^ and 2004–2005.^[13]^ Both Shijiazhuang city and Shexian County participated in the first and second surveys, but Shijiazhuang city missed the third survey. For this study, we are focused on urban-rural comparison of ASMR of cervical cancer between Shijiazhuang city and Shexian County.

### 2.4 Statistical methods

Segi (world) standard population (modified by Doll) was used to calculate ASIRs of cervical cancer.^[14]^ Because we had population-based tumor registration data for Shijiazhuang city only for 2012 and for Shexian County for 2000–2015, urban-rural comparison of ASIR of cervical cancer was performed only for 2012 and the difference between them was tested by the approximate method.^[15]^ The trends of biennial ASIR and averaged age at diagnosis of cervical cancer in Shexian County between 2000 and 2015 were analyzed using Join Point Regression Analysis 4.2.0.2.^[16]^ The number of minimum and maximum join point is set as 0 and 1, and sex and year (biennial) was set as the bivariable and independent variable, respectively.

The study was approved by the Institutional Ethics Review Board of Fourth Hospital of Hebei Medical University.

## 3 Results

### 3.1 Comparison of age-standardized incidence rate of cervical cancer between urban Shijiazhuang city and rural Shexian County in 2012

In 2012, a total of 64 cervical cancer were diagnosed among the 197,416 women in Shexian County, giving a crude incidence rate of 32.1 and an ASIR of 25.0 per 100,000. Cervical cancer accounts for 11.7% of all new cancer cases. It is the third most common cancer in women, after stomach and esophagus cancer (Table 1).

**Table 1:**
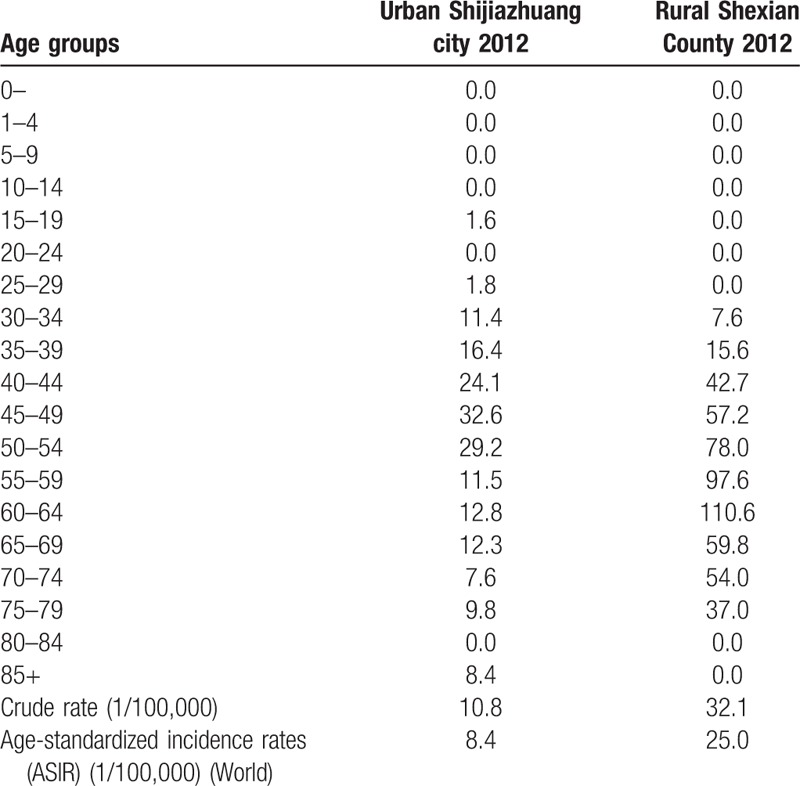
Comparison of age-specific cervical cancer incidence rates between urban Shijiazhuang and rural Shexian County in 2012.

In comparison, a total of 132 cervical cancer cases were diagnosed in Shijiazhuang city among the 1,217,437 registered women, giving a crude incidence rate of 10.8 and an ASIR of 8.4 per 100,000. Cervical cancer accounts for 5.2% of all new cancer cases and is the fifth most common cancer among Shijiazhuang women, after breast, lung, colorectal, and stomach cancers (Table 1).

**Figure 2:**
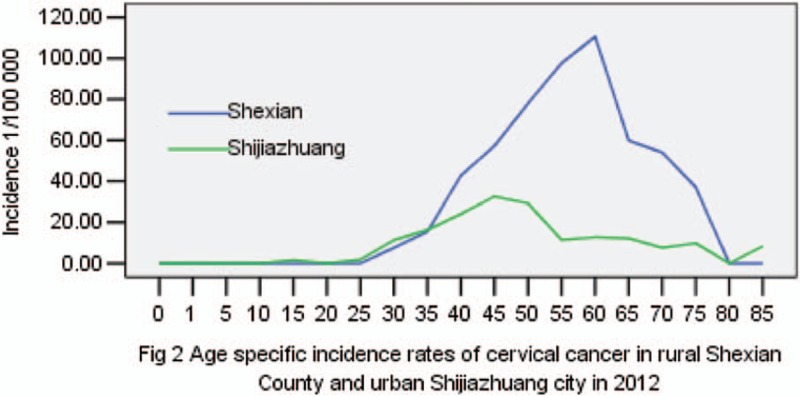
Age-specific incidence rates of cervical cancer in rural Shexian County and urban Shijiazhuang city in 2012.

The ASIR of cervical cancer in rural Shexian County was 3 times higher than in urban Shijiazhuang (25.2 vs. 8.4 per 100,000, *P* < .01). The corresponding cumulative incidence rate of between 0 and 74 years old women is 3.7 times higher (2.98% vs. 0.81%, *P* < .01) (Table 1). As shown in Figure 2, the rural excess in incidence rate of cervical cancer is apparent over the age range between 40 and 80 years old.

### 3.2 Urban-rural disparity in age-standardized mortality rate of cervical cancer between urban Shijiazhuang city and rural Shexian County

In the first Chinese national death survey of the 1973–1975 period, the ASMR of cervical cancer was 14.6 per 100,000 per year nationally.^[10]^ Rural counties in Shanxi province such as Xiangyuan, Yangcheng, and Wuxiang, in Figure 1, were found to have the highest rates of cervical cancer (22.0–54.4 per 100,000 per year), and the rate in the adjoining Shexian County of Hebei province was similarly high (25 per 100,000 per year). However, the ASMR was only half (13.0 per 100,000 per year) in Shijiazhuang city, 250 km northeast of Shexian County (Table 2, Fig. 1).^[11]^ There was a significant urban-rural disparity in cervical cancer mortality in each period. In 1973–1975, the ASMR in rural Shexian was two times higher than in urban Shijiazhuang (25.0^∗^ vs. 13.0^∗^ per 100,000), and the rural excess was 7 to 8 times larger over the 1990–1992 period (10.7^†^ vs. 1.5^†^ or 9.8^∗^ vs. 1.2^∗^ per 100,000 per year) (Table 2).

**Table 2:**
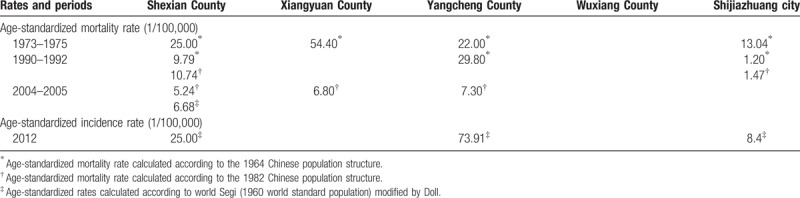
Age-standardized incidence and mortality rates of cervical cancer in Shexian County and Shijiazhuang city and in the adjoining counties in Shanxi province.

### 3.3 Disparity in socioeconomic development and in access to opportunistic cytological screening for cervical cancer between urban Shijiazhuang and rural Shexian

Cytological screening for precancerous cervical lesion by Papanicolaou test is believed to have significantly reduced the incidence rate of invasive cervical cancer.^[1,2]^ In the test, epithelium cells are collected from the surface of the uterine cervix and the smeared cells are cytologically examined directly under a microscope. This kind of tests is recommended only in the Department of Gynecology in city hospitals (opportunistic screening) in China because of a scarcity of cytologists and infrastructure in rural areas. Although starting from a similar rural agrarian background 60 years ago, Shexian County is socioeconomically considerably less developed than Shijiazhuang city (Table 3).^[17,18]^ As reflected in an urban-rural inequity in medical resource allocation, Shexian women have no access to Papanicolaou testing. Most cervical cancer cases diagnosed there are found in late stages after symptoms develop. In contrast, Papanicolaou testing is offered in the outpatient clinic of the Department of Gynecology in every hospital in urban Shijiazhuang city since early 1970s, and it is covered by public medical insurance programs. In addition, adult women employees in Shijiazhuang city are offered a free annual health check-up in which Papanicolaou testing is included (Table 3).

**Table 3:**
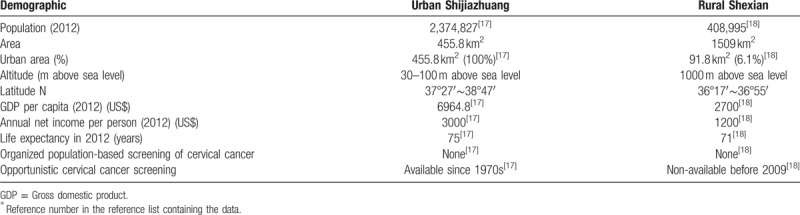
Disparity in socioeconomic development and access to opportunistic cervical cancer screening between urban Shijiazhuang city and rural Shexian County^∗^.

### 3.4 Increasing incidence of cervical cancers and decreasing age at diagnosis in rural Shexian County from 2000 to 2015

**Figure 3:**
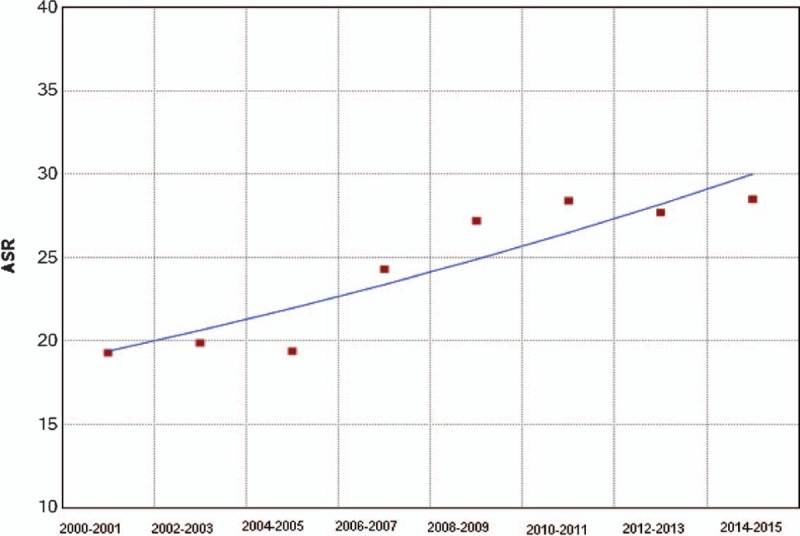
Age-standardized incidence rate (biennial) of cervical cancer in Shexian County from 2000 to 2015; averaged biennial percent change = +3.2%, *P* < .01.

**Figure 4:**
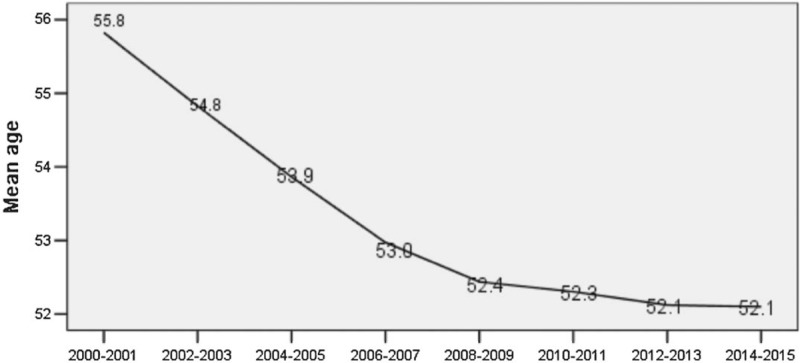
Averaged age at diagnosis (biennial) of cervical cancer in Shexian County from 2000 to 2015.

In trend analysis, the biennial ASIR of cervical cancer in Shexian County increased by 47.7% from 19.3 per 100,000 per year women in 2000–2001 to 28.5 in 2014–2015 (averaged biennial percent change = +3.2%, *P* < .01) (Fig. 3). Meanwhile, the biennial mean age at diagnosis of cervical cancer in Shexian County decreased from 55.8 years old in 2000–2001 to 52.1 in 2014–2015 (*P *< .01) (Fig. 4). Together, these may suggest a cohort effect that later generations of Shexian women are being exposed to a higher risk of cervical cancer or at a younger age.

## 4 Discussion

In the present study, we found rural Shexian has a 3 times higher ASIR and a 2–8 times higher ASMR (degree varying according to various outcome measuring rates) of cervix cancer than urban Shijiazhuang city. This rural excess of cervical cancer is by far the largest ever reported in China. Our findings are in agreement with a meta-analysis.^[19]^ Authors of the meta-analysis had been engaged in decade long international collaborative study on cervical cancer in Shanxi province. By synthesizing mortality rate of cervical cancer from three national mortality surveys and global and national cervical cancer registration data, the meta-analysis concluded that cancer registration data in China have failed to estimate the true incidence rates of cervical cancer for rural China in recent decades, because available population-based tumor registration sites in rural China are usually located at socioeconomically better off areas, and the urban rural difference in ASIR of cervical cancer estimated according to such a sample is too small.

Rural excess of cervical cancer in China may be partially explained by the lack of access to screening. As stated in GLOBOCAN 2012, the large geographic variation in cervical cancer rates reflects differences in the availability of screening, which allows for the detection and removal of precancerous lesions, and human papillomavirus (HPV) infection prevalence.^[1]^ Cervical cytology screening programs offering Papanicolaou testing every 3–4 years have reduced cervical cancer incidence and mortality by up to 80% in USA,^[20]^ England,^[21]^ Hong Kong,^[22]^ Australia, and New Zealand in the past 5 decades.^[23]^ In Hong Kong, though population-based screening of cervical cancer has never been organized, opportunistic cytological screening available since 1970 has successfully decreased both the age-standardized incidence and mortality rates from 1972 to 2001.^[24]^ Although to a much less extent, women living in mainland Chinese cities have benefitted similarly as in Hong Kong from opportunistic cytological screening. Opportunistic screening of cervical intraepithelial neoplasia (CIN) by Papanicolaou testing is covered in Chinese public medical insurance programs and is a ritual practice in the Outpatient Clinic in Department of Gynecology in every city hospital in the mainland. Moreover, a free annual health check-up, including cervical cytological screening, is offered by the employer institutions to all adult urban female employees. However, cytological screening of cervical cancer requires high quality expertise and equipment. Due to urban-rural inequity in medical resource allocation, rural counties in mainland China often do not have a department of cytology or pathology. Before 2009, population-based cytology-screening programs have never been organized among rural women and most of them have never been screened in whole life time. When symptom develops, the cancer is often at late stage. In summary, extremely limited access to cervical cancer screening may have caused rural women having a higher burden of cervical cancer.

Shexian women not only have a 3 times higher ASIR of cervical cancer than Shijiazhuang women, but the incidence is increasing in recent decade. China is undergoing rapid changes in sexual mores. There is increased acceptance of premarital sex and extramarital sex, especially among youth. Hu et al^[3]^ reported that an increasing trend in both incidence and mortality of cervical cancer was observed in mainland China in either rural or urban area since 2000. This is in contrast to the trend in Hong Kong where ASIR of cervical cancer decreased significantly from 24.9 in 1972–1974 to 9.5 in 1999–2001 per 100,000 per year women.^[24]^ Both urban women in mainland China and Hong Kong women have had access to opportunistic pap smear screening; therefore, the increase in mainland China suggests that the effect of changing sex behavior in parallel with socioeconomic development and urbanization is greater than the incidence decreasing effect of opportunistic screening.

Though rural women have significantly higher incidence and mortality rates of cervical cancer than their urban counterpart in China, appropriate action or program has not been taken yet by government or health authorities. The reason for this lacking of action may be that policy makers think, like other cancers, the etiology of cervical cancer is complicated and its prevention is too expensive and difficult. But these are false perceptions.

Although cytology-based screening programs are not feasible in resource-limited rural areas because they need complex human, financial, and bricks-and-mortar infrastructures to sustain, secondary prevention is possible in socioeconomically less developed areas, with alternative approaches to the costly cytological method used in developed countries, as recently recommended by WHO.^[25]^ For example, visual inspection with acetic acid (VIA) to screen for cervical cancer is very cost-effective if a screen-and-treat approach is used. This newly suggested procedure suggests that women are screened and treated at the same visit. The sensitivity and specificity of VIA in detecting high-grade cervical intraepithelial neoplasia grade 2 and 3 lesions are 50% and 85%, respectively.^[26]^ The feasibility, safety, acceptability of a single-visit approach with VIA and cryotherapy have been shown in Thailand, and in several African countries^[27–30]^; and the effect has been approved by randomized trials in India.^[31–33]^

In addition to screening, primary prevention by implementing HPV vaccination as part of national immunization programs is more cost-effective. Compared with screening, HPV vaccination provides long-term protection, requires few visits, generates herd immunity, and protects against persistent HPV16 and HPV18 infections.^[34–36]^

Previous urban-rural classification of cancer registries in China was made according to the administration level.^[37]^ If it is a prefecture-level city (province governing city) or above, it is urban; if it is a county or county-level city, it is rural. However, a typical Chinese prefecture-level city also includes several rural counties. When these people are misclassified as urban, the real urban-rural difference is diluted. This may partly explain the contradictory results previously reported about urban-rural disparity in cancer for China.^[38]^ With regard to this, we restricted comparison between a pure urban city and a rural county.

A limitation may be asked about the representativeness of Shexian-Shijiazhuang disparity to represent China; because Shexian County has been an endemic region for esophagogastric and cervical cancers, it may be socioeconomically more disadvantaged than other counties in China and it may have exaggerated urban-rural disparity in infection-related cervical cancer. However, situated in northern central China, Shexian County is socioeconomically more developed than most counties in southwestern and northwestern China. In 2010, it had a total product value of 21.4 billion YMB yuan (3.6 billion USA$). Its economy power ranked 19th during 2001–2005 and 13th during 2006–2010 among 136 counties in Hebei province.^[18]^ Moreover, urban-rural disparity in cancer in China is not limited to a rural excess of incidence or mortality of infection-related cancer. It is apparent also in the survival rate. Zeng et al^[39]^ calculated the age-standardized 5-year relative survival for all cancers combined and 26 individual cancers types for 138,852 new cancer cases diagnosed between 2003 and 2005 in 17 population-based cancer registries in China. They found that the age-standardized 5-year relative survival for rural patients was only about half that of their urban counterparts for all cancers combined (21.8% vs. 39.5%, *P *< .01); this pattern was similar for all individual cancer types except for esophageal cancer, and the difference was statistically significant for 22 cancer types, including cervical cancer. This striking disparity in cancer survival ensures us that urban-rural disparity in cancer in China is so pervasive that Shijiazhuang-Shexian difference presented here is by no means an outlet.

In summary, we found a rural excess of 3 times higher in ASIR and 2–8 times higher in ASMR with cervical cancer in Hebei province, China. Rural-urban disparity in cancer is found systemically from incidence to mortality to survival rates. The age-standardized 5-year relative survival rate was significantly poorer with rural cancer patients than their urban counterparts for all cancer combined and for most individual cancer types except for esophageal cancer (with which patients in rural area showed a significantly improved survival than their urban counterpart) in China^[39]^ suggests the effect of increased population-based endoscopic screening of esophageal cancer and mucosal resection programs in rural endemic areas since 2000s.^[40]^ Similarly, tackling the rural excess of cervical cancer is feasible now through population-based screening by VIA and treatment through a single-visit screening and treat approach in rural endemic areas. However, fighting the growing risk of exposure to HR-HPV infection in China as suggested by increasing incidence and decreasing averaged age at diagnosis should be through primary prevention with HPV vaccination. Vaccines against HPV are a great scientific advance. They must be incorporated into national immunization program to adolescent girls in China. Prompt action is most important.

## 5 Conclusion

Urban-rural disparity in ASIR of cervical cancer in present study is larger than that reported between developed and less developed countries in GLOBOCAN 2012, in which the disparity is considered “due to differences in access to screening.” As in China, cytologists and infrastructure required for cervical cancer screening are similarly lacking in rural areas, we suggest cytological screening for cervical cancer be strengthened in disadvantaged rural settings.

## Author contributions

**Conceptualization:** Denggui Wen, Yunjiang Liu.

**Data curation:** Denggui Wen, Yi Yang, Yuetong Chen, Kohei Akazawa, Baoen Shan, Yunjiang Liu.

**Formal analysis:** Denggui Wen.

**Funding acquisition:** Denggui Wen.

**Investigation:** Xiaoduo Wen, Denggui Wen, Yi Yang, Yuetong Chen, Kohei Akazawa, Baoen Shan, Yunjiang Liu.

**Methodology:** Xiaoduo Wen, Kohei Akazawa.

**Resources:** Xiaoduo Wen.

**Writing – original draft:** Xiaoduo Wen, Denggui Wen, Yi Yang, Kohei Akazawa, Baoen Shan.

**Writing – review & editing:** Xiaoduo Wen, Denggui Wen.

Denggui Wen orcid: 0000-0002-5557-1965.
